# Life Cycle Assessment to Quantify Global Warming and Human Health–Respiratory Impacts of Using Composites from Waste Wind Turbine Blades as Feedstock for Cement Clinker and Fiberglass Production

**DOI:** 10.1021/acs.est.5c07978

**Published:** 2025-10-02

**Authors:** Caroline V. Cameron, Sabrina Spatari, Jason B. Baxter, Megan A. Creighton

**Affiliations:** † Department of Chemical and Biological Engineering, 6527 Drexel University, Philadelphia, Pennsylvania 19104, United States; ‡ Faculty of Civil and Environmental Engineering, 26747 Technion - Israel Institute of Technology, Haifa, 3200003, Israel

**Keywords:** life cycle assessment, circular economy, wind energy, wind turbine blade, fiber reinforced polymer composite, end-of-life, cement coprocessing, pyrolysis

## Abstract

The wind energy sector is a growing contributor to global electricity generation. The increasing deployment of wind turbines also creates significant waste when turbine materials reach their end-of-life. Glass fiber reinforced polymer composites, which comprise the majority of a wind turbine blade’s mass, are difficult to separate into their component parts for recycling. This study employs a cradle-to-gate life cycle assessment to evaluate the environmental impacts of utilizing waste wind turbine blade material in cement clinker and fiberglass production. We find that incorporating waste blades as 15% of the feedstock in a cement clinker production plant reduces global warming and human health–respiratory impacts by 9 and 34%, respectively, compared to using virgin materials only. For a fiberglass plant, this substitution increases global warming impacts by 11% but decreases respiratory health impacts by 3%. Each kilogram of secondary product diverts approximately 0.25–0.32 kg of WTB waste from landfills. The projected rate of blade decommissioning of ∼800,000 tonnes per year would replace less than 1% of the overall virgin material demand for the cement clinker industry and up to 8% for the fiberglass industry, indicating plenty of capacity for these industries to accommodate this waste blade material in their feedstocks.

## Graphical abstract

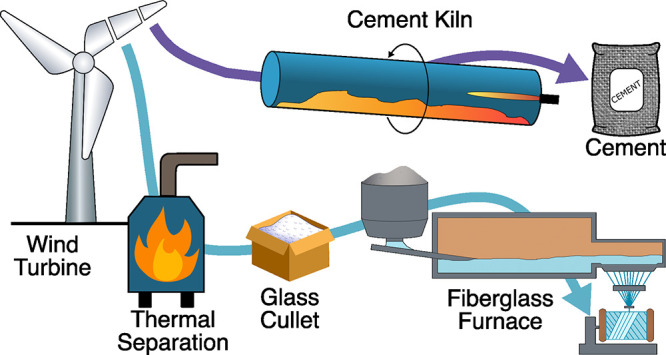

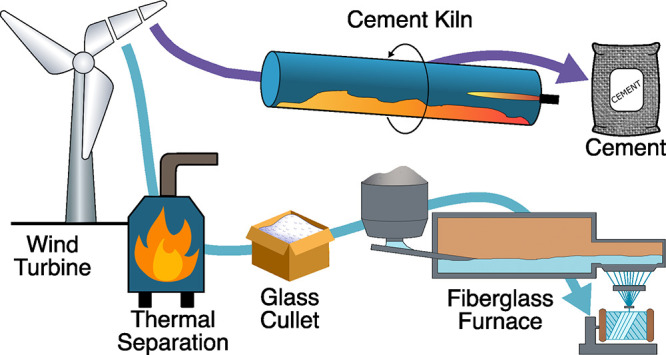

## 1 Introduction

Wind energy accounted for 6.7% of global electricity in 2021,^1,2^ and installations are projected to double in the coming years.^3^ However, growth in this sector also generates an increasing stream of solid waste as the wind turbine blades (WTBs) are decommissioned after approximately 20 years of service life. A global total of 13–43 million tonnes (t) of decommissioned blade material is expected by 2050.^4–6^ These blades are made of about 85% fiber reinforced polymer composites (FRPC) by mass, with smaller quantities of foams, adhesives, and wood^7–9^ (see inset in Figure 1 outlined in black). 65–90% of FRPC in the wind industry exclusively uses glass fibers; the balance are a hybrid that include carbon FRPC in specific segments of the blade to increase strength and reduce weight, enabling longer blades and higher power turbines.^10,11^ Glass FRPC comprises a thermoset epoxy matrix^11,12^ and either borosilicate glass called E-glass or a boron-free variation with improved chemical resistance and mechanical properties known as E-CR glass.^13,14^ These glasses are low in cost, high in strength and stiffness, and provide chemical resistance.^14,15^ Recent efforts to design for recycling focus on thermoplastic resin systems,^16,17^ alternate thermoset chemistries with dynamic covalent bonds,^18^ or carbon fiber composites.^19–21^ These emerging material technologies represent significant advances for the future of waste recovery.^6,12,22,23^ However, most blades that will be decommissioned in the next few decades will be made from epoxy-glass composites, and technical solutions to address this growing solid waste stream are needed.

**Figure 1:**
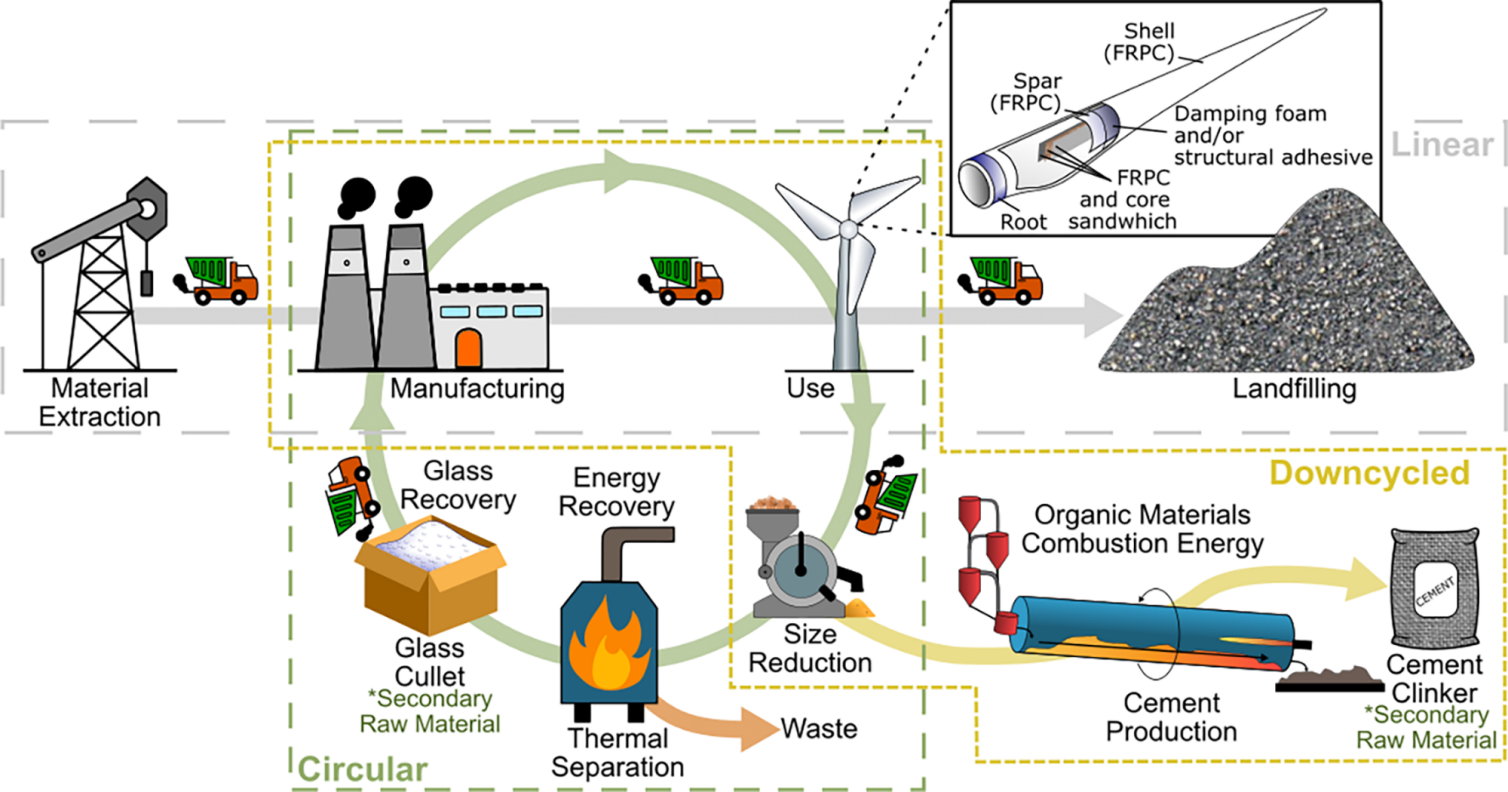
Life cycle stages of linear (gray), downcycled (yellow), and circular (green) life cycles. The downcycled and circular life cycles produce secondary raw materials cement clinker and glass cullet, respectively. The inset figure shows the structure, components, and materials commonly found in a wind turbine blade.

Linear, downcycled, and circular options for decommissioned glass composite-based WTBs are in various stages of development. Currently, direct disposal to landfill is the dominant pathway in the US due to a lack of established alternatives for glass FRPC (see gray system boundary in Figure 1). Direct recycling of the individual components in a WTB is challenging because of the strong interfacial adhesion between the nonrecyclable thermoset polymer and the fiberglass, which inhibits their separation.^24^ Chemical recycling has not been implemented at scale,^12,25–27^ but there are multiple pyrolysis facilities that process blade waste to recover the reinforcing fibers and produce a pyrolysis fuel.^4,21,28^ Alternatively, WTB composites can be diverted from landfill by downcycling into lower-value products through minimal segmentation to repurpose in parks, bridges, or roofs,^12,29–31^ or recycled by mechanical grinding for use in injection-molded paneling or filler.^19,23,32,33^ Repurposing pathways only divert a small amount of material from the landfill and are limited by scalability to process all waste WTB. Waste disposal in a cement kiln has been implemented in Europe,^4,34^ and a cement coprocessing blade recycling program in the US was started in 2020 by General Electric and Veolia.^35,36^

In this work, we systematically evaluate the environmental impacts of two industrially available strategies for converting composite waste from WTBs into secondary raw materials: cement clinker, produced by cement coprocessing, and glass cullet recovered from pyrolysis (Figure 1, yellow and green outlines). Although the wind industry only accounts for 10–24% of glass and carbon FRPC in the composites industry,^37,38^ wind farms provide a highly concentrated point source of waste composites from the blades. The proposed alternative end-of-life (EoL) options can be piloted on the wind waste stream and later extended to address glass FRPC materials from other sectors including construction, automotive, maritime, or defense.^14^

In typical cement clinker production, raw materials (e.g., limestone, sand, bauxite, *etc.*) are extracted, ground, and fed into a preheating chamber. They are heated to ∼900 °C and release carbon dioxide (CO_2_) as the virgin materials thermally decompose to their oxide forms (calcium oxide, silica, alumina, etc*.*).^39,40^ The materials then enter the kiln, where they are heated to >1400 °C for 20–60 min and react further to form the cement clinker product. Cement coprocessing is the use of waste materials as alternative fuels and raw materials in cement kilns during clinker production. This process enables simultaneous energy recovery and material recycling, as the high temperatures ensure complete combustion and incorporation of inorganic residues into the final product. When waste blades are used in cement coprocessing, the glass portion of the blade serves as an alternative feedstock of oxides in the cement clinker, which reduces the demand for virgin raw materials and the associated direct CO_2_ emissions from the decomposition reactions. Additionally, the organic materials in the waste blade combust within the preheater and provide a source of localized heating, which offsets a portion of fossil fuels required for standard kiln operation.^34,41^

Pyrolysis is a thermal treatment performed in an inert environment to suppress standard combustion reactions of organic materials while promoting production of amorphous carbon char and high-energy hydrocarbon molecules (e.g., methane, ethane, etc*.*).^21,42^ These high-energy molecules can be collected as pyrolysis fuels and combusted as an alternative heat source to offset natural gas.^21^ The organic polymers in the blades thermally decompose between 300–800 °C.^21,42^ These temperatures are below the melting point of glass, allowing the fibers to be recovered, although they are mechanically weakened and entangled,^21,41^ limiting the applications for direct reuse. The recovered fiberglass can be additionally purified by thermal oxidation to remove amorphous carbon char that could participate in side reactions that create defects in the next glass product.^43,44^ The purified fiberglass can then be used as a cullet feedstock to the glass furnace, substituting for 15–30% of virgin materials.^45–47^

The raw materials in a standard glass batch react at 1400 °C for 10–15 h to produce the final glass composition, releasing CO_2_ in the process.^15,48,49^ Cullet utilization offsets virgin materials needed to manufacture glass, reduces direct CO_2_ emissions from decomposition reactions, and reduces energy demand to operate the glass furnace.^46,50–53^ Additionally, remelting glass removes defects and enhances its mechanical properties.^54^ Currently, cullet utilization in E-glass production is minimal in the glass industry due to strict manufacturing requirements,^51,53^ which could limit this alternative EoL pathway. However, in-house cullet (i.e., manufacturing scrap) is already used in fiberglass production,^53^ which indicates the potential for fiberglass cullet implementation, if the recovered cullet is clean from contaminants and has a compatible composition. If E-glass fibers can be used as cullet to form new fibers and manufactured into new blades, then both the wind and glass industries could move toward a more circular economy.

Prior analyses have demonstrated that cement coprocessing and pyrolysis are technically viable EoL solutions for WTBs.^21,34,41,55^ A techno-economic analysis by Ghosh et al. indicates that the cost of cement coprocessing is not a barrier to the transition toward a more circular EoL for WTBs in the US.^56^ Additionally, the variable operational cost of cement coprocessing is low, around $0.18 per kilogram of recycled material, due to low capital equipment needs to implement the process in the cement industry.^20^ The cost of virgin E-glass fibers is low ($1–3 per kilogram), while the cost of epoxy resin is up to twice as expensive.^20,57^ The variable operational cost of pyrolysis is greater than that of cement coprocessing, at about $0.22 per kilogram of recycled material.^20^ However, the environmental impacts on the cement and glass industries resulting from the use of waste blades as feedstock require greater attention. In particular, analyses of pyrolysis have identified the challenges of strength loss and entanglement in fiber recovery^13,21,43,58^ but have not assessed the impacts of recovering the fiberglass as cullet for recycling into glass manufacturing.

In this work, we use attributional life cycle assessment (LCA) to quantify the environmental impacts of producing cement clinker and fiberglass with blended fractions of FRPC from waste WTBs to replace raw materials, and we benchmark these pathways with conventionally produced cement clinker and fiberglass without waste feedstock integration. We consider the energy and material inputs and emissions of greenhouse gases (CO_2_eq) and fine particulate matter (PM_2.5_, <2.5 μm). Scenarios with waste feedstock assume 15% of feed to the cement kiln or fiberglass furnace comes from waste WTBs. Below we describe the goal and scope, life cycle inventory, life cycle impact assessment, and interpretation—including sensitivity analyses—of LCA results for a case study in the Midwest region of the United States.

## 2 Methods

A cradle-to-gate attributional LCA was used to compare the environmental and energy impacts of cement clinker and fiberglass production with and without waste WTB material input following the ISO 14040/44 standards.^59,60^ We treat the retired WTB as waste and develop an inventory for size-reducing and transporting the waste feedstocks to cement and pyrolysis processing facilities. We use system expansion rules to credit offset raw materials used to produce the WTB-*co*-blended cement clinker and fiberglass products. Thus, the burden to produce a WTB was not included in this analysis, as the blades were a waste material. Bottom-up mass and energy balances were used to calculate material and energy quantities and model the process flows for each product, based upon literature values related to material compositions, operation energy requirements, and emission factors. Thermogravimetric analysis was performed to experimentally determine the combustion temperature and production of amorphous carbon char during pyrolysis of organic materials in the WTB.

The functional unit was defined as 1 kg of secondary material (cement clinker or fiberglass). An alternative functional unit of 1 kg of waste WTB considered by others^19,20^ does not facilitate comparison of cement clinker and fiberglass with and without waste feedstock. We used SimaPro PhD Release 9.1.0.7 and the ecoinvent V3 database to apply the TRACI 2.1 life cycle impact assessment (LCIA) method for the US to assess the midpoint environmental burdens of the secondary material production pathways.^61,62^ Global warming (GW) and human health–respiratory (HHR) impacts were included in this inventory, measured by carbon dioxide equivalents (CO_2_eq) and PM_2.5_ emissions, respectively. These impact categories were selected because significant CO_2_eq and PM_2.5_ emissions result from both cement clinker and glass production processes. While other TRACI 2.1 impact categories are also important, we focused on these two categories because of their relevance to high-temperature processes and relative availability and degree of certainty in the data.

### 2.1 Goal and Scope

The goal of this LCA was to determine the environmental impacts of utilizing waste WTB material as a feedstock in the cement and glass industries. A waste input rate of 15% of the feedstock to a cement kiln and fiberglass furnace was selected to reflect typical waste and cullet input rates in cement coprocessing and glass manufacturing plants.^40,63–66^ In cement coprocessing, the waste fed to the kiln included both organic and inorganic (i.e., glass) content from the waste blade. In fiberglass manufacturing, the waste fed to the glass furnace comprised only the recovered glass cullet, excluding organic content in the waste blade. The feed of each virgin material was varied independently, based upon the composition of the waste glass, to achieve a target clinker or glass composition. The system boundaries for this analysis are shown in Figure 2. The burden to produce wind turbine blades was not included in this analysis, as the blades were a waste material. WTB decommission and metals recovery were out-of-scope for this analysis.

**Figure 2:**
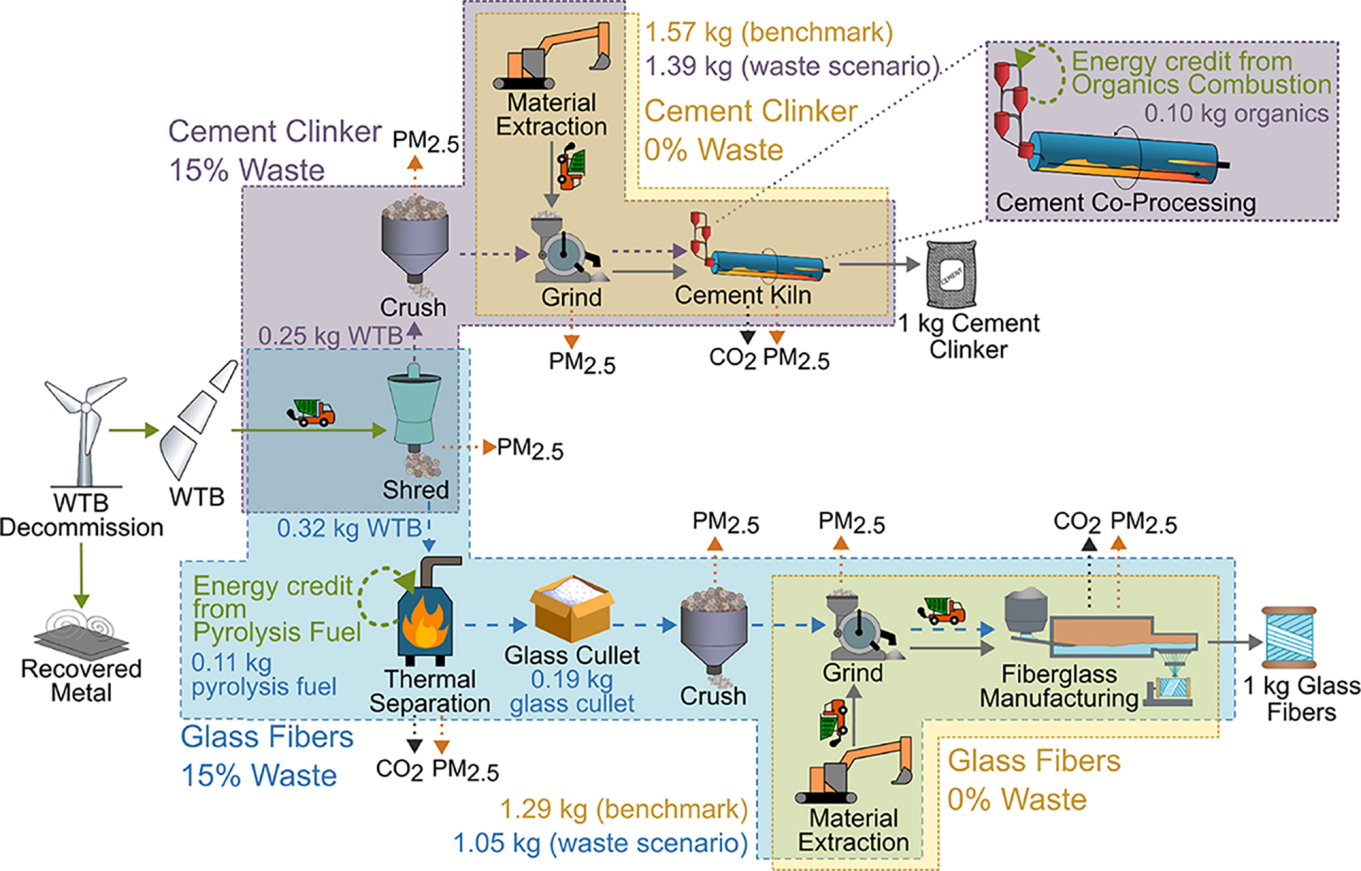
System boundaries for the process steps to produce cement clinker and fiberglass using only virgin raw materials (yellow) or a mixture of virgin and waste-derived materials (purple/teal).

The majority of onshore wind turbines in the US (∼61.5%) are located in the Midwest;^67^ therefore, this analysis includes the Midwest Reliability Organization (MRO) electric grid and processing facilities generally located in Missouri. The locations of facilities and quarries, along with the distances between them used in the analysis, are detailed in Tables S1 and S2. A pyrolysis facility does not currently exist in Missouri, so the location of the Carbon Rivers facility in Tennessee was used. A sensitivity analysis on these transportation distances is provided in Section 3.2. Details of assumptions and the approach to develop the life cycle inventory are provided below.

### 2.2 Life Cycle Inventory

Baseline inventories for the LCA were developed with consideration of material, energy, and transportation inputs, CO_2_eq and PM_2.5_ emissions, and energy credits for each process step within the system boundaries. The compositions of E-glass and cement clinker are provided in Table 1. Virgin materials to produce cement clinker included limestone, sand, bauxite, iron ore, magnesite, and soda ash. For borosilicate E-glass production, kaolin was chosen as the source of alumina rather than bauxite and boric acid was the source of boron oxide, according to common industry practice.^15,53,68^ Kaolin provides a second source of silica in addition to virgin sand. Both virgin materials were considered in the calculation of the mass of silica in the final fiberglass product. Thermal decomposition reactions of these virgin materials are shown in Table 2.

**Table 1:** Material Compositions for E-Glass and Cement Clinker in the Baseline Benchmark and Waste Scenarios^a^

		cement clinker (%)	
component	E-glass (%)	benchmark	15% waste
CaO	21.5	65.0	64.5
SiO_2_	56.0	21.5	21.3
Al_2_O_3_	14.2	6.5	6.5
Fe_2_O_3_	0.2	3.5	3.5
MgO	2.5	3.0	3.0
Na_2_O	0.6	0.5	0.5
B_2_O_3_	5.0	-	0.7

a The compositions of E-glass^15,53,71^ and the benchmark cement clinker^34,71^ were sourced from literature. The cement clinker waste scenario composition was calculated using mass balances (see SI) and thermal decomposition reactions of the virgin materials (see Table 2). The composition of E-glass was consistent between the recovered cullet and the benchmark and waste scenarios.

**Table 2:** Virgin Material Sources and Their Thermal Decomposition Reactions

virgin material	thermal decomposition reaction
limestone	CaCO_3_ → CaO + CO_2_
sand	SiO_2_ → SiO_2_
bauxite	Al_2_O_3_·2H_2_O → Al_2_O_3_ + 2H_2_O
kaolin	Al_2_Si_2_O_5_(OH)_4_ → Al_2_O_3_ + SiO_2_ + 2H_2_O
iron ore	Fe_2_O_3_ → Fe_2_O_3_
magnesite	MgCO_3_ → MgO + CO_2_
soda ash	Na_2_CO_3_ → Na_2_O + CO_2_
boric acid	2H_3_BO_3_ → B_2_O_3_ + 3H_2_O

In the waste scenarios, the oxides in the waste glass offset a portion of virgin materials. For example, calcium oxide (CaO) was present in the composition of both cement clinker and recovered fiberglass; the mass of CaO in the glass portion of the waste blade contributed to the total mass of CaO in the final composition of the clinker. The remaining mass of CaO in the final clinker product was then sourced from limestone. Similar considerations were made for the other components of the clinker and glass products. Boron oxide is a component of E-glass that is not in a standard cement clinker composition. In the waste feedstock scenario, we targeted the same compositional ratios in the waste-free clinker product while also accounting for boron oxide from E-glass in the waste blade. The modified composition of the clinker was assumed to have no effect on quality, as noted in prior research by Andersen et al.^4^ and Sproul et al.^20^ Following literature guidance, the alternative fuel for cement coprocessing should contain less than 0.2% chlorine to avoid adverse effects on cement clinker quality.^69^ Polyvinyl chloride (PVC) is used in some models of blades,^7,9^ but it was not present in the blade composition for this analysis, which represents more common material choices.^8,34,57,70^ Coprocessing plants receiving blades containing PVC may need to use a ratio of waste to virgin feed of less than 15%.

We assumed that cement coprocessing required 3.5 MJ per kilogram of clinker produced,^72^ including energy for the preheater, precalciner, and kiln, with a fuel mix of 75% coal and 25% natural gas based upon the coal-heavy mixes reported by other analyses on cement clinker production.^20,63,72^ This energy input represents the total required to form the clinker phase and was assumed to be the same in both the benchmark and waste scenarios. Clinker formation reactions were assumed to be unchanged between scenarios and were not explicitly modeled. In the waste feedstock scenario, the masses, heat capacities, and combustion temperatures of each material in the waste blade were used to calculate the energy to heat these materials in the preheater. Net heats of combustion,^73–75^ less an assumed 15% loss,^72^ were used to determine the energy credit from combusting them within the preheater (see Table S3). We assumed that the energy credit had the same fuel mix as the cement kiln. The kiln operation energy in the waste feedstock scenario was less than that in the benchmark scenario because: (1) there was a lower mass of virgin materials in the waste scenario, and (2) the organic materials in the waste blade only had to be heated to their combustion temperatures, which were lower than the temperatures of the preheater or the kiln. Complete combustion reactions of the organic materials in the waste blade were considered to calculate the mass of CO_2_ emitted by combustion of these materials in the waste scenario.

We assumed that the fiberglass furnace required 15.0 MJ per kilogram of fiberglass produced and used a fuel mix of 76% natural gas and 24% electricity; electricity is used to power batch preparation and fiber forming steps, and natural gas is used as heat for melting, refining, and postforming process steps within glass manufacturing.^15^ In the waste feedstock scenario, pyrolysis recovers glass cullet from the waste blade and produced amorphous carbon char and pyrolysis fuel. An oxidation step removed the carbon char from the recovered cullet by reacting it with oxygen to form CO_2_. Pyrolysis and oxidation required 5.65 MJ per kilogram of feed material^21^ and were heated by natural gas. The pyrolysis fuel was collected and burned as an alternative heat source in the pyrolysis reactor. The pyrolysis fuel mix analyzed by Coughlin et al. was used to model pyrolysis fuel in this analysis (Table S4); it had an energy content of 16.5 MJ per kilogram and provided an energy credit for natural gas.^21^

Due to the differences in clinker and glass production processes, and the focus in this analysis on a consistent quantity of product, the amounts of waste WTB fed to these processes differed. With a waste input rate of 15%, the cement industry could process 0.245 kg of waste WTB per kilogram of clinker produced, while the glass industry could process 0.316 kg of waste WTB per kilogram of fiberglass produced (Table S5). This may be surprising, given that these waste quantities are greater than 15% of the 1 kg functional unit. However, the waste utilization rate applies to the feedstock into the cement kiln and fiberglass furnace, not the final products. As such, material losses due to gaseous byproducts factor into the total quantities of waste blade material that enter each process.

Size reduction process steps were modeled by a gyratory crusher, cone crusher, and ball mill.^76^ Particulate matter emissions were calculated using emission factors^77,78^ and material flow for each process step (Table S6 and Figure S1).

## 3 Results and Discussion

### 3.1 Life Cycle Impact Assessment

We present TRACI 2.1 midpoint results for global warming and human health–respiratory impacts from the use of 15% waste WTB feedstock in cement clinker and fiberglass production, benchmarked relative to WTB-free production pathways.

#### 3.1.1 Global Warming Impact Results

There are two types of CO_2_ equivalent emissions within our system boundaries: direct emissions resulting from chemical reactions (e.g., thermal decomposition, combustion, and oxidation) during the process steps, and indirect emissions resulting from the operation energy to power the process steps. Direct CO_2_eq emissions were calculated using the thermal decomposition and complete organic material combustion reactions and are provided in Table S7. Indirect emissions were calculated through ecoinvent database selections and the TRACI 2.1 impact method. Allocated CO_2_eq emissions from each process step are provided for each product scenario in Figure 3.

**Figure 3:**
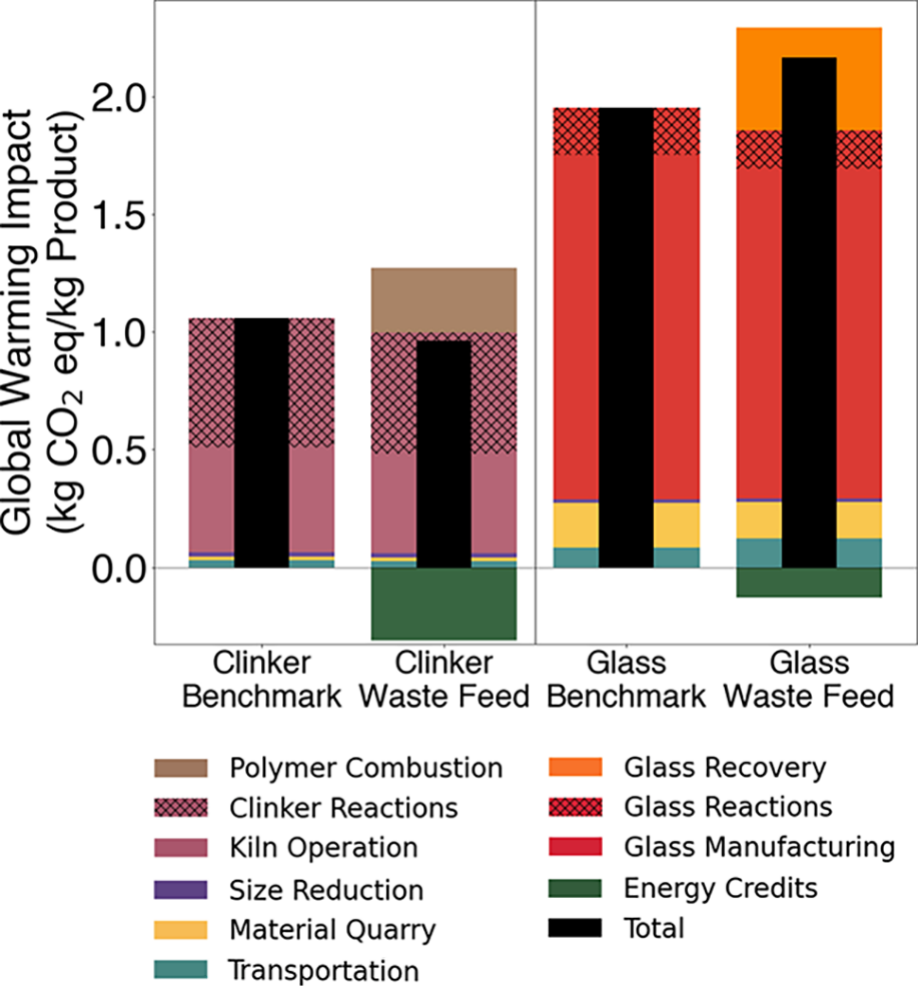
Global warming impacts for cement clinker and fiberglass production with and without waste WTB feedstock. Impacts are shown as relating to transportation (teal), material quarrying and extraction (yellow), size reduction (purple), kiln operation (mauve), polymer combustion (brown), pyrolysis (orange), glass manufacture (red), and energy credits (green). The black bars indicate net totals of all inputs, outputs, and credits.

In the benchmark scenario, we calculated 1.06 kg CO_2_eq emissions per kilogram of cement clinker produced (black bar in Figure 3), which is slightly greater than other literature estimates of 0.7–1.0 kg CO_2_eq.^63,79,80^ This impact range can largely be attributed to variations in kiln operation energy and specific fuel mixes.^20,72^ Kiln operation energy and CO_2_eq emissions from the decomposition reactions of virgin materials in the kiln contributed 94% of the total GW in this benchmark scenario. Virgin material quarrying, size reduction, and transportation contributed relatively little GW by comparison.

The GW from producing 1 kg cement clinker with 15% waste WTB input was 0.96 kg CO_2_eq, which was about 9% less than our benchmark. This reduction in emissions for the waste scenario aligned with results from other analyses.^20,34^ When including waste WTB in the feedstock, indirect CO_2_eq emissions from kiln operation energy were 4% less than the benchmark scenario due to a reduction in the amount of virgin materials that entered and reacted within the kiln. Additionally, direct CO_2_eq emissions were 6% less than the benchmark scenario because portions of the CaO, MgO, and other components in the clinker product were sourced from the glass in the waste blade rather than virgin materials. Combustion of the polymeric materials in the waste WTB within the kiln resulted in direct CO_2_eq emissions, which accounted for 29% of the total GW impact. However, the local heating from this combustion offset some of the heat from fossil fuels and was therefore considered an energy credit to the system.

The GW to produce 1 kg of fiberglass in the conventional benchmark process was 1.95 kg CO_2_eq (Figure 3), which was within the range of other literature estimates of 1.7–2.5 kg CO_2_eq^61,81–83^ 10% of the GW in this benchmark scenario was from quarrying virgin materials. 75% of the GW was from glass manufacturing, due to the high temperature and long residence time of the glass melt stage,^48^ and from use of the coal-heavy MRO electric grid to supply 24% of the furnace operation energy.^1,61^ 10% of the GW impact resulted from direct emissions by decomposition reactions and 4% resulted from transportation. The long transportation distances for raw materials—particularly boric oxide sourced from Boron, California—resulted in a transportation component of GW that was nearly five times greater for fiberglass than for cement clinker.

Fiberglass manufacturing using 15% glass cullet recovered from waste WTB generated 2.17 kg CO_2_eq emissions—11% greater than the benchmark scenario (Figure 3). Use of cullet reduced GW from virgin material extraction by 19%, direct CO_2_eq emissions from decomposition reactions by 18%, and indirect emissions from glass manufacturing by 4% compared to the benchmark scenario. Additionally, a natural gas energy credit was applied to account for the offset from energy generated by combusting the pyrolysis fuels. However, the pyrolysis and oxidation processes needed to recover and clean the glass cullet—along with emissions from pyrolysis fuel combustion—contributed 20% of total GW emissions in the fiberglass waste scenario, ultimately outweighing the upstream benefits. This overall increase in GW emissions with pyrolysis aligned with findings by Sproul, et. al.^20^

#### 3.1.2 Human Health–Respiratory Impact Results

Fine particulate matter (PM_2.5_) is emitted during fuel combustion, size reduction processing, and cement kiln and fiberglass furnace operation. A removal efficiency of 95% of PM_2.5_ emissions, typical of standard industry filtration practices, was applied to direct particulate emissions from the in-scope process steps.^84–88^ Direct PM_2.5_ emissions from the size reduction processes were minimal compared to those from other process steps due to the low PM_2.5_ emission factors; note that larger particulate sizes were out of scope for this analysis.^89^ The direct PM_2.5_ emissions from each process step are provided in Table S7, and the allocated HHR impacts for each production scenario are shown in Figure 4.

**Figure 4:**
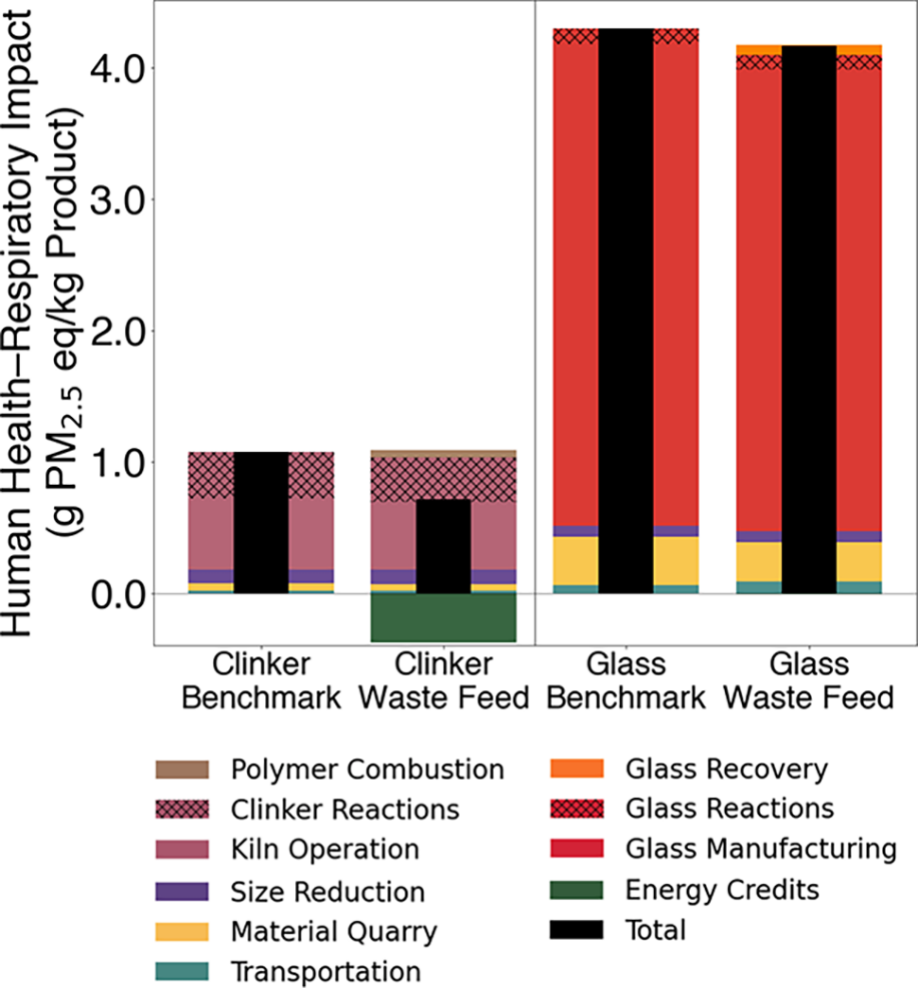
Human health–respiratory impacts for cement clinker and fiberglass production with and without waste WTB feedstock. Impacts are shown as relating to transportation (teal), material quarrying and extraction (yellow), size reduction (purple), kiln operation (mauve), polymer combustion (brown), pyrolysis (orange), glass manufacture (red), and energy credits (green). The black bars indicate net totals of all inputs, outputs, and credits.

For cement clinker, the HHR impact dropped by 33.6%, from 1.07 to 0.71 g PM_2.5_eq per kilogram, primarily due to the energy credit from polymer combustion. Coal has a high PM_2.5_ emission factor (0.2 g per MJ), while natural gas emits significantly less (0.01 g per MJ).^61^ In the cement coprocessing scenario, 75% of the 2.4 MJ of energy produced from polymer combustion offsets coal use and just 25% offsets natural gas, leading to a substantial reduction in PM_2.5_ emissions. This overall PM_2.5_ reduction mirrors the GW trends observed between the benchmark and waste feedstock scenarios.

For glass production, the HHR impact declined more modestly (by 3%, from 4.29 to 4.16 g PM_2.5_eq per kilogram) when waste WTBs were used. Though the reduction was small, it was still meaningful, especially given that PM_2.5_ emissions in glass production are inherently higher than in clinker production due to the greater operation energy.

When recovered cullet was incorporated, PM_2.5_ emissions from virgin material quarrying dropped by 19%, and those from glass manufacturing decreased by 4% compared to the benchmark scenario. These reductions outweighed the additional emissions introduced during the cullet recovery process (pyrolysis, oxidation, and combustion of pyrolysis fuels), resulting in a net benefit for HHR. The pyrolysis fuel energy credit was modeled entirely as natural gas, which has a lower PM_2.5_ emission factor compared to coal. Additionally, the pyrolysis fuel provided an energy credit of 1.9 MJ, which was lower than that in the cement clinker waste scenario. As a result, the avoided PM_2.5_ emissions were lower in the glass waste scenario than in the cement clinker waste scenario.

For context on the scale of the emissions presented in this section, a standard passenger vehicle emits 0.2 kg CO_2_eq and 0.039 g PM_2.5_ per km driven.^1,90^ These CO_2_eq and PM_2.5_ emission factors equate the manufacture of 1 kg of cement clinker (or fiberglass) to driving about 5 (10) km and 20 (110) km, respectively.

### 3.2 Sensitivity Analyses

Sensitivity analyses were performed on the rate of waste incorporation, glass composition, and transportation requirements. Results are shown in Figure 5 and discussed below.

**Figure 5:**
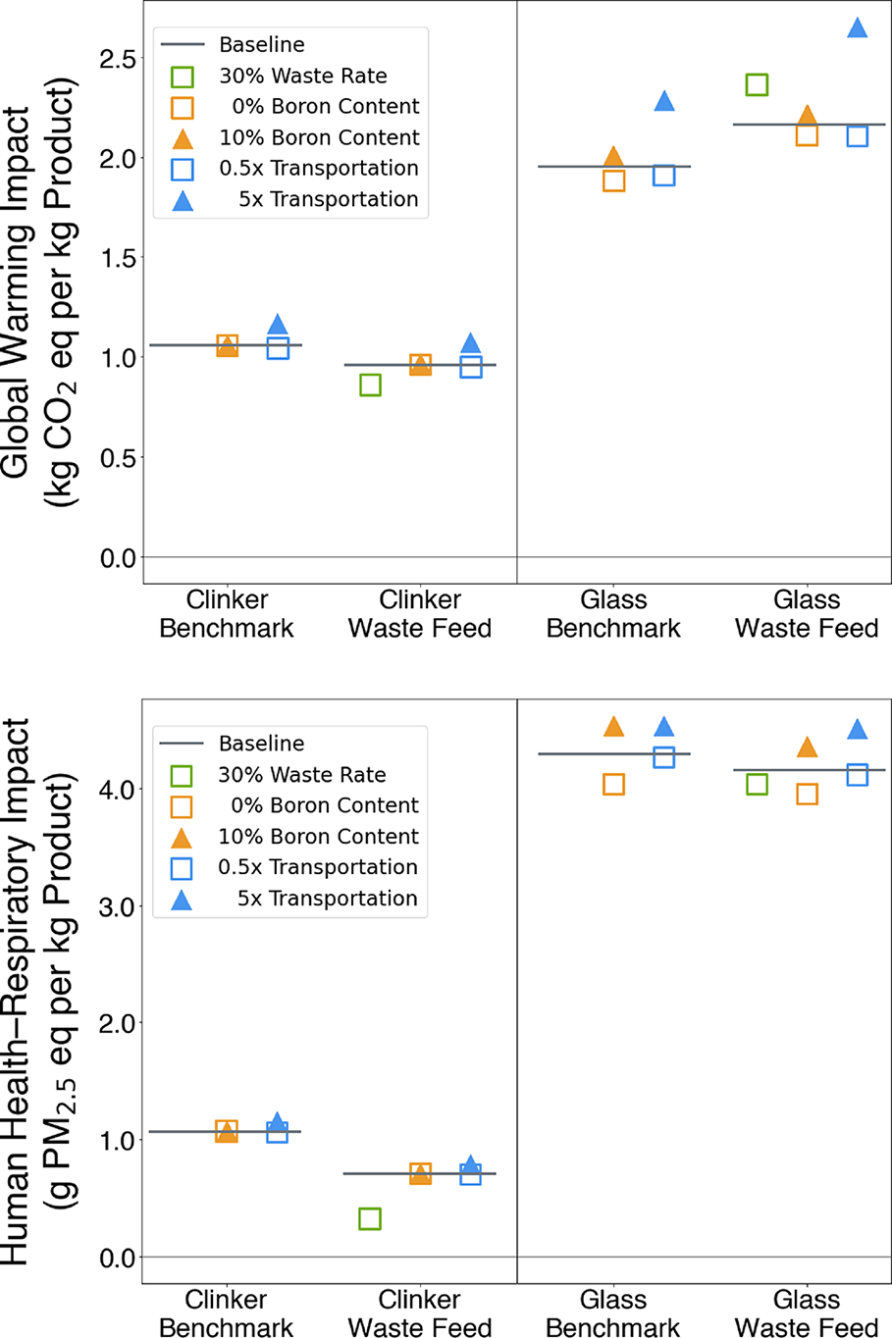
Sensitivity results for waste rate set at 30% (green), glass composition (orange), and transportation (blue) compared against the baseline results (horizontal bars) for GW (top) and HHR (bottom).

The rate of waste incorporation was 15% in the baseline analysis to represent a realistic waste input rate used in cement coprocessing and a common cullet input rate used in glass manufacturing.^40,63–66^ This fraction may increase as recycling in cement clinker and fiberglass manufacturing matures. Therefore, we doubled the waste incorporation rate to 30% waste feedstock to the kiln and fiberglass furnace in the sensitivity analysis to represent more mature recycling in both industries. The same trends observed between the benchmark and waste feedstock scenarios for clinker and fiberglass held true as the waste rate increased.

The composition of E-glass can vary depending on the desired application and the specific manufacturer.^15,50,91,92^ The composition of B_2_O_3_ in the final glass can range from 0 to 10% of the total composition;^15^ a composition of 0% B_2_O_3_ represents E-CR glass rather than E-glass. In recent years, the fiberglass industry has been adopting E-CR glass in place of E-glass due to its improved chemical resistance and mechanical strength.^14^ For the baseline scenario, the B_2_O_3_ composition was set at 5%, and a sensitivity analysis explored variations within the 0–10% range; the glass compositions tested in the sensitivity analysis are provided in Table S8. The impacts from cement clinker production were insensitive to the boron content of the glass fibers in the WTB. However, it is important to limit the addition of boron in clinker, as its presence can slow cement cure times.^34^ The GW and HHR impacts from both fiberglass production scenarios increased as B_2_O_3_ content increased due to the higher impact associated with boric acid. Fiberglass production with no B_2_O_3_ content and no waste in the feedstock resulted in the lowest GW impact out of all fiberglass production scenarios tested because it avoided the higher impacts from boric acid and did not have additional emissions from glass recovery.

Transportation accounted for 5% or less of the total GW and HHR impacts for both the cement clinker and fiberglass production processes, which was much less than direct and other indirect emissions from these processes. When the transportation distance was decreased by a factor of 50% compared to the baseline requirement to represent improvements in the supply chain, it accounted for 3% or less of GW and HHR impacts for the tested scenarios. When this distance increased by a factor of 5x to represent poorer supply chain infrastructure, it accounted for 8–23% of GW and HHR impacts.

## 4 Prospects for WTB Circularity

This work considers two waste utilization pathways for WTBs with mature technology readiness: cement coprocessing and pyrolysis coupled with glass cullet recovery and remanufacturing. These options divert waste blades from the landfill and increase material circularity from the wind industry. We assumed the blades are primarily made of an epoxy polymer matrix that is reinforced with fiberglass. The fiberglass content can offset typical virgin materials in cement clinker and fiberglass production, while combustion of the organic portion of the blades can provide an energy credit to offset standard fuel mixes to power a cement kiln and pyrolysis reactor. Increasing the incorporation of glass cullet from waste blades into fiberglass production increased GW, which indicates that the GW benefits of using cullet in fiberglass manufacturing may not outweigh the GW emissions from recovering clean cullet with current methods and supply chains. In contrast, increasing the incorporation of waste blades into cement clinker production reduced GW and HHR impacts.

To increase circularity, there must be an alignment between availability and demand in material supply chains. The rate of decommissioned WTBs could reach 800,000 t/year globally by 2050,^19^ with a cumulative total of 13–43 million tonnes.^4–6^ The cement industry produced ∼4.1 billion tonnes of cement globally in 2020,^93^ and the fiberglass industry produced ∼9 million tonnes globally in 2023.^94^ Given these annual production rates, the projected rate of blade decommissioning would account for much less than 1% of the total feedstock to the cement industry and up to 8% of the total feedstock to the fiberglass industry, indicating ample capacity for both industries to accommodate blade waste. Additionally, adoption of coprocessing at a cement plant requires little to no adjustments to cement kilns as they exist and operate today.^20^

This work provides quantitative insight into the environmental impacts of incorporating composites from waste WTBs in cement clinker and fiberglass manufacturing. While WTBs were used as an example, similar logic applies to other sectors of the composites industry. We provide a data-driven impact assessment that should be combined with consideration of economics, material properties, and supply chain logistics to design sustainable waste-to-resource pathways across interconnected industries.

## Supplementary Material





## Notes

Additional details about model inputs: facility locations; material compositions; material and energy quantities; database selections; and detailed calculations (PDF)
Excel file used to develop life cycle inventor (XLSX)
