# Supplementary material for: Life Cycle Assessment to Quantify Global Warming and Human Health–Respiratory Impacts of Using Composites from Waste Wind Turbine Blades as Feedstock for Cement Clinker and Fiberglass Production
Source: Environ Sci Technol. 2025 Oct 2;59(40):21476–85. doi: 10.1021/acs.est.5c07978 (PMC12529953; doi:10.1021/acs.est.5c07978)
Supplement: Supplementary file 1 [file es5c07978_si_001.pdf]

Supporting Information Available

Life Cycle Assessment to Quantify Global Warming and Human Health–Respiratory Impacts of Using Composites from Waste Wind Turbine Blades as Feedstock for Cement Clinker and Fiberglass Production

Supplementary Information

Caroline V. Cameron†

Sabrina Spatari‡

Jason B. Baxter†[1]

Megan A. Creighton†[2]

†Department of Chemical and Biological Engineering, Drexel University, Philadelphia, PA, 19104, USA

‡Faculty of Civil and Environmental Engineering Technion - Israel Institute of Technology, Haifa, Israel, 3200003

[1]Email: jbaxter@drexel.edu

[2]Email: mc4298@drexel.edu

Additional Figures, tables, and calculations are provided. Figures include a detailed process flow diagram of the system boundaries with greater detail of material quantities and a graphical display of the energy requirements for each process step in the baseline benchmark and waste scenarios. Tables include locations for processing facilities and virgin material quarries used in the analysis; corresponding transportation distances between facilities used in the analysis; material composition and properties for a glass FRPC wind turbine blade; material composition and properties of pyrolysis fuel; a summary of material, energy, and transportation inputs calculated for the baseline benchmark and waste scenarios; operation energy and fine particulate matter emission factors for equipment used in the analysis; direct carbon dioxide and fine particulate matter emissions from each process step for the baseline benchmark and waste scenarios; a summary of compositions for fiberglass used in the glass composition sensitivity analysis; and a summary of life cycle inventory ecoinvent database selections. Equations include those used to calculate virgin material inputs in the benchmark and waste scenarios; carbon dioxide emissions from thermal decomposition reactions, organic material combustion, and pyrolysis fuel combustion; operation energy requirements for the cement kiln and fiberglass furnace; and energy generated from combustion of organics. The Excel workbook used to develop the life cycle inventory is also included.

12 pages

2 figures

10 tables

10 equations

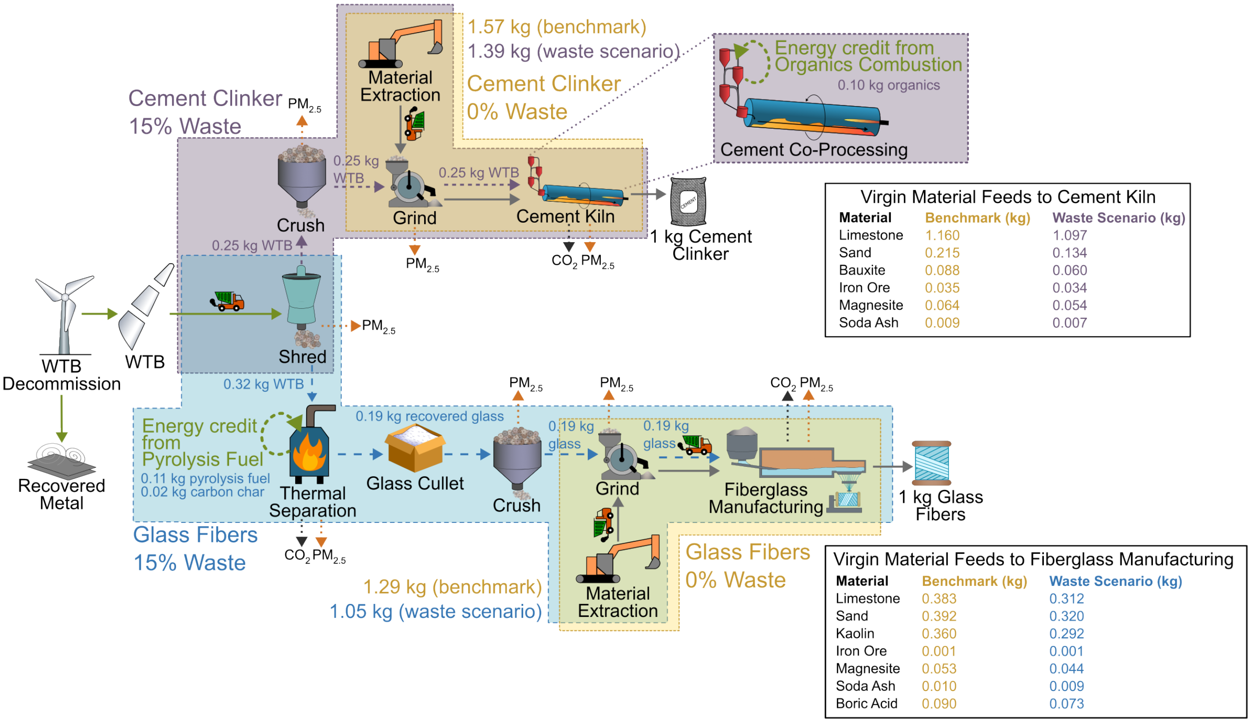

Figure S1: A detailed process flow diagram of the clinker and fiberglass production processes, including mass quantities.

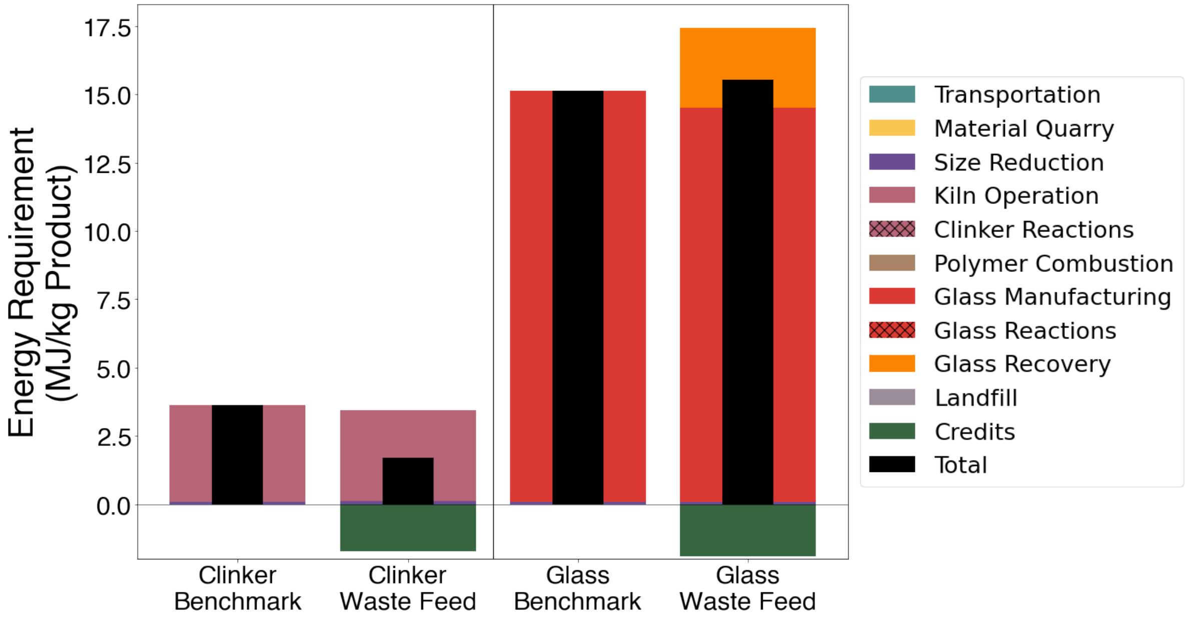

Figure S2: The operation energy inputs are indicated as positive values, while the energy credits are indicated as negative values.

Table S1: Addresses for processing facilities and virgin material quarries considered in analyses.

Facility Name	Address
Wind farm	8479 State Hwy E, Union Star, MO 64494
Landfill	9431 50th Rd SE, St Joseph, MO 64507
Cement plant	2200 N Courtney Rd, Sugar Creek, MO 64050
Pyrolysis facility	2409 Sycamore Dr, Knoxville, TN 37921
Glass manufacturing facility	1415 E Boonville St, Sedalia, MO 65301
Limestone quarry	21008 Unity Ave, Gallatin, MO 64640
Sand quarry	4401 N Cobbler Rd, Independence, MO 64058
Bauxite quarry	17850 W Sardis Rd, Bauxite, AR 72011
Kaolin quarry	520 Kaolin Rd, Sandersville, GA 31082
Iron ore quarry	17113 Co Rd 58, Nashwauk, MN 55769
Magnesite quarry	17113 Co Rd 58, Nashwauk, MN 55769
Soda ash quarry	13210 Cameron Rd, Excelsior Springs, MO 64024
Boric acid quarry	Boron, CA 93516

Table S2: Transportation distances between facilities and quarries.

Starting Facility	Ending Facility	Distance (km)
**Cement Clinker Transportation**		
Wind farm	Cement plant	130
Limestone quarry	Cement plant	130
Sand quarry	Cement plant	20
Bauxite quarry	Cement plant	650
Iron ore quarry	Cement plant	1015
Magnesite quarry	Cement plant	1015
Soda ash quarry	Cement plant	40
**Fiberglass Transportation**		
Wind farm	Pyrolysis facility	1260
Pyrolysis facility	Glass manufacturing	1085
Limestone quarry	Glass manufacturing	190
Sand quarry	Glass manufacturing	150
Kaolin quarry	Glass manufacturing	1420
Iron ore quarry	Glass manufacturing	1120
Magnesite quarry	Glass manufacturing	1120
Soda ash quarry	Glass manufacturing	150
Boric acid quarry	Glass manufacturing	2620

Table S3: Material properties of a wind turbine blade. The distribution of materials in a WTB was based upon an average of values reported in the literature.[7–9,70] Char production represents the conversion of each organic material to amorphous carbon char after pyrolysis; these values were experimentally determined via thermogravimetric testing at 10 °C/minute to 600°C in an inert nitrogen gas environment. Combustion temperature was also determined via thermogravimetric analysis and represents the temperature at the onset of rapid thermal decomposition for each organic material. Heats of combustion for each organic material in the WTB were found in literature.

Material	Literature Composition (%)	Composition in analysis (%)	Char Product (%)	Combustion Temperature (°C)	Heat of Combustion (MJ/kg)
E-glass	53.8–64	58.7			
Epoxy	13–36.8	24.8	6.1	350	32.5[73]
Foam	1–16.9	6.0	26.2	260	24[73]
PU	0–6.5	3.5	20.7	330	6.69[75]
PE	0–10	3.0	22.6	350	47.74[73]
Wood	0–5.3	4.0	18.0	250	14.8[74]

Table S4: The composition, density, and molecular weight of pyrolysis fuel, as analyzed by Coughlin, et. al.[21]

Component	Volumetric Composition
Carbon monoxide	19.1%
Carbon dioxide	12.0%
Methane	27.6%
Ethane	2.2%
Ethene	3.0%
Propane	0.4%
Propene	1.1%
i-Butane	0.02%
n-Butane	0.05%
Hydrogen	26.5%
Oxygen	1.2%
Nitrogen	5.2%
Density	0.9 kg/m^3
Molecular Weight	20.0 g/mol

Table S5: Material, energy, and transportation inputs per kilogram of secondary product, derived using bottom-up mass and energy balances.

		Cement Clinker		Fiberglass	
Process steps	Units	Benchmark	15% Waste	Benchmark	15% Waste
**Virgin material inputs**					
Limestone	kg	1.160	1.097	0.383	0.312
Sand	kg	0.215	0.134	0.392	0.320
Bauxite	kg	0.088	0.060	-	-
Kaolin	kg	-	-	0.360	0.292
Iron ore	kg	0.035	0.034	0.001	0.001
Magnesite	kg	0.064	0.054	0.053	0.044
Soda ash	kg	0.009	0.007	0.010	0.009
Boric acid	kg	-	-	0.090	0.073
Waste WTB	kg	0	0.245	0	0.316
**Energy inputs**					
Size reduction	MJ	0.101	0.107	0.083	0.082
Cement kiln	MJ	3.52	3.33	-	-
Pyrolysis	MJ	-	-	-	1.78
Oxidation	MJ	-	-	-	1.14
Glass furnace	MJ	-	-	15.04	14.41
**Transportation requirements**					
Virgin materials	kgkm	313	274	941	765
Blade waste	kgkm	0	32	0	398
Recovered glass	kgkm	-	-	0	201
**Energy credits**					
Coal	MJ	-	1.833	-	-
Natural gas	MJ	-	0.611	-	1.886

Table S6: Energy and direct $PM_{2.5}$ emission factors used in the analysis.

Factor	Value	Units	Reference
**Shredder (gyratory crusher)**			
Operation Energy (electricity)	5.3×10^{-3}	MJ/kg feed	[76]
$PM_{2.5}$ Emissions	-	kg/kg feed	[89]
**Crusher (cone crusher)**			
Operation Energy (electricity)	1.1×10^{-3}	MJ/kg feed	[76]
$PM_{2.5}$ Emissions	5×10^{-8}	kg/kg feed	[89]
**Grinder (ball mill)**			
Operation Energy (electricity)	6.5×10^{-2}	MJ/kg feed	[76]
$PM_{2.5}$ Emissions	3×10^{-8}	kg/kg feed	[89]
**Cement Kiln**			
(Operation energy sourced from 75% coal and 25% natural gas[20])			
Operation Energy	3.5	MJ/kg clinker	[72]
$PM_{2.5}$ Emissions	3×10^{-4}	kg/kg feed	[84]
**Organic Material Combustion**			
$PM_{2.5}$ Emissions	1.1×10^{-2}	kg/kg feed	[77]
$PM_{2.5}$ Filtration Efficiency	95%		[78]
**Fiberglass Furnace**			
(Operation energy sourced from 76% natural gas and 24% electricity[15])			
Operation Energy	15.0	MJ/kg fiberglass	[15]
$PM_{2.5}$ Emissions	1.2×10^{-4}	kg/kg fiberglass	[15]
**Pyrolysis Reactor**			
Operation Energy (natural gas)	5.65	MJ/kg feed	[21]
**Oxidation Reactor**			
Operation Energy (natural gas)	5.65	MJ/kg feed	[21]
**Recovered Pyrolysis Fuel Combustion**			
Energy Credit (natural gas)	16.55	MJ/kg fuel	[21]
$PM_{2.5}$ Emissions	1.1×10^{-2}	kg/kg fuel	[77]
$PM_{2.5}$ Filtration Efficiency	95%		[78]

Table S7: Direct carbon dioxide and fine particulate emissions per kg of secondary product.

		Cement Clinker		Fiberglass	
Process steps	Units	Benchmark	15% Waste	Benchmark	15% Waste
**Carbon dioxide**					
Size Reduction	kg	-	-	-	-
Kiln system	kg	0.547	0.514	-	-
Polymer Combustion	kg	-	0.275	-	-
Oxidizer	kg	-	-	-	0.060
Combustor	kg	-	-	-	0.178
Glass Furnace	kg	-	-	0.200	0.164
**Fine particulate matter**					
Size Reduction	g	5.49×10^{-5}	6.93×10^{-5}	4.51×10^{-5}	5.25×10^{-5}
Kiln system	g	3.51×10^{-1}	3.42×10^{-1}	-	-
Polymer Combustion	g	-	5.40×10^{-2}	-	-
Fuel Combustor	g	-	-	-	6.10×10^{-2}
Glass Furnace	g	-	-	1.16×10^{-1}	1.11×10^{-1}

Table S8: Percent compositions of compounds in fiberglass used in the glass composition sensitivity analysis.[15]

Compound	Minimum Boron	Baseline	Maximum Boron
CaO	23.0	21.5	19.0
SiO_2	57.0	56.0	55.2
Al_2O_3	14.7	14.2	14.0
Fe_2O_3	0.5	0.2	0.3
MgO	4.0	2.5	1.0
Na_2O	0.8	0.6	0.5
B_2O_3	0	5.0	10.0

Table S9: EcoInvent database selections used to represent material, transportation, and energy inputs.

<table>
<tr><th>Inventory Input</th><th>Model Input</th></tr>
<tr><td>Materials</td><td></td></tr>
<tr><td>Limestone</td><td>Limestone, unprocessed RoW| limestone quarry operation | Cut-off, U</td></tr>
<tr><td>Sand</td><td>Sand RoW| sand quarry operation, extraction from river bed | Cut-off, U</td></tr>
<tr><td>Silica sand</td><td>Silica sand RoW| production | Cut-off, U</td></tr>
<tr><td>Bauxite</td><td>Bauxite GLO| bauxite mine operation | Cut-off, U</td></tr>
<tr><td>Kaolin</td><td>Kaolin RoW| production | Cut-off, U</td></tr>
<tr><td>Iron ore</td><td>Iron ore, crude ore, 46% Fe GLO| iron mine operation, crude ore, 46% Fe | Cut-off, U</td></tr>
<tr><td>Magnesite</td><td>Dolomite RoW| production | Cut-off, U</td></tr>
<tr><td>Soda ash</td><td>Soda ash, dense GLO| modified Solvay process, Hou's process | Cut-off, U</td></tr>
<tr><td>Boric acid</td><td>Boric acid, anhydrous, powder RoW| production | Cut-off, U</td></tr>
<tr><td>Transportation</td><td></td></tr>
<tr><td>Truck</td><td>Transport, freight, lorry >32 metric ton, EURO6 RoW| transport, freight, lorry >32 metric ton, EURO6 | Cut-off, U</td></tr>
<tr><td>Energy</td><td></td></tr>
<tr><td>Coal</td><td>Heat, district or industrial, other than natural gas RoW| heat production, at coal coke industrial furnace 1-10MW | Cut-off, U</td></tr>
<tr><td>Natural gas</td><td>Heat, district or industrial, natural gas RoW| heat production, natural gas, at industrial furnace >100kW | Cut-off, U</td></tr>
<tr><td>Electricity</td><td>Electricity, low voltage MRO, US only| market for | Cut-off, U</td></tr>
</table>

Calculations

Table S10: Variable definitions for the calculations used to develop the Life Cycle Inventory.

Variable	Definition
$m_{material}$	Input mass of material
$w_{material,product}$	Mass fraction of material in the final product
P	Mass of clinker or fiberglass produced
$MW_{material}$	Molecular weight of material
$\nu_{material}$	Stoichiometric coefficient of material in decomposition reaction
$\rho_{material}$	Density of material
q_{loss}	The heat loss factor for the cement kiln

Equations 1 and 2 are examples of the method used to calculate the mass of virgin materials input to the cement kiln or fiberglass furnace and the mass of carbon dioxide emitted by the chemical decomposition reactions. Table S10 defines the variables in these equations.

$$m_{limestone} = w_{CaO,\ product} \times P \times \frac{MW_{limestone}}{MW_{CaO}} \times \frac{\nu_{limestone}}{\nu_{CaO}} \quad (1)$$

$$m_{CO_2} = w_{CaO,\ product} \times P \times \frac{MW_{CO_2}}{MW_{CaO}} \times \frac{\nu_{CO_2}}{\nu_{CaO}} \quad (2)$$

Equation 3 shows the method used to calculate the mass of sand required to produce E-glass. This calculation accounts for the amount of silica sourced from recovered glass cullet in the waste blade and silica that is produced from thermal decomposition of kaolin.

$$m_{sand} = \left[w_{SiO_2,glass} \times P - m_{SiO_2,blade} - \left(m_{kaolin} \times \frac{MW_{SiO_2}}{MW_{kaolin}} \times \frac{\nu_{SiO_2}}{\nu_{kaolin}} \right) \right] \times \frac{MW_{sand}}{MW_{SiO_2}} \times \frac{\nu_{sand}}{\nu_{SiO_2}} \quad (3)$$

Equation 4 was used to calculate the mass of CaO in the glass portion of a waste blade. Equation 5 was used to calculate the mass of limestone still required to produce the desired

composition of CaO in the product. The calculation method shown in these equations was also used for the other components of the product compositions.

$$m_{\mathrm{CaO,blade}} = w_{\mathrm{CaO,glass}} \times w_{\mathrm{glass,blade}} \times m_{\mathrm{blade}} \tag{4}$$

$$\begin{aligned} m_{\mathrm{limestone}} = &\ (w_{\mathrm{CaO,product}} \times \mathrm{P} - m_{\mathrm{CaO,\ blade}}) \\ &\times \frac{\mathrm{MW}_{\mathrm{limestone}}}{\mathrm{MW}_{\mathrm{CaO}}} \times \frac{\nu_{\mathrm{limestone}}}{\nu_{\mathrm{CaO}}} \end{aligned} \tag{5}$$

Equation 6 was used to calculate the mass of CO_2 that is emitted by combustion of the organic materials in the blade. This combustion occurs in the cement kiln in the waste feedstock scenario for clinker production. The subscript i represents each of the organic materials in the blade.

$$m_{\mathrm{CO_2,comb}} = m_{\mathrm{blade}} \times \sum_i \left(\frac{\mathrm{MW}_{\mathrm{CO_2}}}{\mathrm{MW}_i} \times \frac{\nu_{\mathrm{CO_2}}}{\nu_i} \right) \tag{6}$$

Equation 7 was used to calculate the mass of CO_2 that is emitted by the combustion of the pyrolysis fuel. The density of the fuel (ρ_{fuel}) and volumetric fractions of each component in the fuel (v_j) were sourced from experimental measurements by Coughlin, et. al.[21]

$$m_{\mathrm{CO_2,fuel}} = m_{\mathrm{fuel}} \times \frac{1}{\rho_{\mathrm{fuel}}} \times \sum_j \left(v_j \times \rho_j \times \frac{\mathrm{MW}_{\mathrm{CO_2}}}{\mathrm{MW}_j} \times \frac{\nu_{\mathrm{CO_2}}}{\nu_j} \right) \tag{7}$$

Equation 8 was used to calculate the operation energy required for the cement kiln. The feed-basis operation energy factor for the kiln ($\hat{\mathrm{E}}_{\mathrm{kiln}}$) was multiplied by the mass of virgin materials that enter the kiln. Then, the energy required to heat the materials in the waste blade was calculated using the mass of the blade materials, their heat capacities, the temperature difference to heat the materials to their combustion temperature (or the temperature of the kiln in the case of glass), and the heat loss (assumed to be 15% for the kiln[72]). As above, the subscript i represents the materials in the blade.

$$E_{\mathrm{kiln}} = m_{\mathrm{v}} \times \hat{E}_{\mathrm{kiln}} + m_{\mathrm{blade}} \sum_{i} \left[w_i C_{\mathrm{p},\, i} \left(T_{\mathrm{comb},i} - T_{\mathrm{RT}} \right) \right] \times (1 + q_{\mathrm{loss}}) \tag{8}$$

Equation 9 was used to calculate the energy to operate the glass furnace.

$$E_{\mathrm{furnace}} = (m_{\mathrm{v}} + m_{\mathrm{glass}}) \times \hat{E}_{\mathrm{furnace}} \tag{9}$$

Equation 10 was used to calculate the energy produced from combustion of the organic materials in the waste blade in the waste feedstock scenario for clinker production. The heat of combustion of each material and the heat loss of 15%[72] were used in this calculation.

$$E_{\mathrm{comb}} = m_{\mathrm{blade}} \sum_{i} \left[w_i \hat{H}_{\mathrm{comb},\, i} \right] \times (1 - q_{\mathrm{loss}}) \tag{10}$$
